# ELMO1 protects renal structure and ultrafiltration in kidney development and under diabetic conditions

**DOI:** 10.1038/srep37172

**Published:** 2016-11-16

**Authors:** Krishna Rakesh Sharma, Karl Heckler, Sandra J. Stoll, Jan-Luuk Hillebrands, Katharina Kynast, Esther Herpel, Stefan Porubsky, Marlies Elger, Boris Hadaschik, Karen Bieback, Hans-Peter Hammes, Peter P. Nawroth, Jens Kroll

**Affiliations:** 1 Department of Vascular Biology and Tumor Angiogenesis, Center for Biomedicine and Medical Technology Mannheim (CBTM), Medical Faculty Mannheim, Heidelberg University, Mannheim, Germany; 2 Division of Vascular Oncology and Metastasis, German Cancer Research Center (DKFZ-ZMBH Alliance), Heidelberg, Germany; 3 Department of Pathology and Medical Biology, Division of Pathology, University Medical Center Groningen, Groningen, The Netherlands; 4 Institute of Pathology, Heidelberg University, Heidelberg, Germany; 5 Tissue Bank of the National Center for Tumor Diseases (NCT), Heidelberg, Germany; 6 Institute of Pathology, Medical Faculty Mannheim, Heidelberg University, Mannheim, Germany; 7 Institue of Neuroanatomy, Medical Faculty Mannheim, Heidelberg University, Mannheim, Germany; 8 Department of Urology, Heidelberg University Hospital, Heidelberg, Germany; 9 Institute of Transfusion Medicine and Immunology and FlowCore Manneim, Medical Faculty Mannheim, Heidelberg University, Mannheim, Germany; 10 Fifth Medical Department, University Medical Centre Mannheim, Mannheim, Germany; 11 Department of Medicine I and Clinical Chemistry, Heidelberg University, Heidelberg, Germany

## Abstract

Engulfment and cell motility 1 (ELMO1) functions as a guanine exchange factor for Rac1 and was recently found to protect endothelial cells from apoptosis. Genome wide association studies suggest that polymorphisms within human *elmo1* act as a potential contributing factor for the development of diabetic nephropathy. Yet, the function of ELMO1 with respect to the glomerulus and how this protein contributes to renal pathology was unknown. Thus, this study aimed to identify the role played by ELMO1 in renal development in zebrafish, under hyperglycaemic conditions, and in diabetic nephropathy patients. In zebrafish, hyperglycaemia did not alter renal ELMO1 expression. However, hyperglycaemia leads to pathophysiological and functional alterations within the pronephros, which could be rescued via ELMO1 overexpression. Zebrafish ELMO1 crispants exhibited a renal pathophysiology due to increased apoptosis which could be rescued by the inhibition of apoptosis. In human samples, immunohistochemical staining of ELMO1 in nondiabetic, diabetic and polycystic kidneys localized ELMO1 in glomerular podocytes and in the tubules. However, ELMO1 was not specifically or distinctly regulated under either one of the disease conditions. Collectively, these results highlight ELMO1 as an important factor for glomerular protection and renal cell survival via decreasing apoptosis, especially under diabetic conditions.

Diabetes mellitus is known to cause several micro- and macrovascular complications within patients worldwide. Diabetic nephropathy is a very concerning microvascular complication of diabetes mellitus, whose global incidence is on the rise, rapidly^1^,^2^. It has been established that diabetic nephropathy eventually progresses into end-stage renal disease and is the leading cause for renal replacement therapy in developed countries^3^. To treat patients suffering from diabetic nephropathy, it is important to understand the underlying mechanisms that lead to the pathology of the disease. It is known that diabetic nephropathy is predominantly associated with increasing proteinuria, which is characterized by the expulsion of proteins such as albumin in the urine, as well as a reduced blood-filtration rate. Diabetes induced albuminuria is initiated primarily due to the loss and/or dysfunction of podocytes^4^. Remarkably, the progression of diabetic nephropathy has been shown to correlate with the loss of podocytes in patients suffering from the microvascular complication^5^. Hyperglycaemia is also known to induce and subsequently increase apoptosis within renal glomerular cells, albeit via the activation of endothelial thrombomodulin–protein C system, which contributes to the pathogenesis of diabetic nephropathy^6^. Hence, this study evidentially demonstrates, that hyperglycaemia induces an increase in apoptosis within the human glomerular endothelial cells and podocytes. However, a better insight must be obtained with respect to the detrimental effects of increased apoptosis in the renal glomerular cells and the subsequent onset of diabetic nephropathy.

Genome wide association studies (GWAS) indicate that polymorphisms in a multitude of genes were associated with the heightened incidence of nephropathy in diabetic patients^7^. To develop new preventative and therapeutic strategies, these polymorphisms need to be studied carefully. Particularly, single nucleotide polymorphisms (SNPs) in human *elmo1* are strongly suggested to be a potential contributing factor for the development of diabetic nephropathy, in various global populations^8^,^9^,^10^,^11^. The ELMO1/DOCK180 complex functions as a guanine nucleotide exchange factor for the small GTPase Rac1, thereby regulating cell migration^12^. ELMO1 is expressed in the vasculature in zebrafish and loss-of-function experiments identified an important role for ELMO1 in early developmental vascular processes^13^. Recently, ELMO1 was shown to protect endothelial cells from apoptosis via activation of the Rac1/PAK/AKT signalling cascade *in vitro* and *in vivo*^14^. ELMO1 has also been implicated in the development of ciliopathy-related phenotypes in zebrafish, by regulating Ezrin phosphorylation^15^. However, the functional role of ELMO1 in human and zebrafish kidneys, with respect to diabetic nephropathy, is currently unknown and is in the need to be established.

The zebrafish is a versatile model organism used to carry out research on various biomedical fields, including diabetes induced complications within the vasculature^16^. The renal system within a zebrafish embryo consists of a pronephros which, although simple in structure, functions to maintain homeostatic balance of ion and metabolite concentrations, and removal of toxic substances in the blood, similar to the human nephron^17^. Thus, this study aims to establish the functional role of ELMO1 in the zebrafish pronephric glomerulus, under hyperglycaemic conditions. We found that, in the zebrafish pronephros, ELMO1 protects the glomerulus from apoptosis and from hyperglycaemia induced damage. These findings are also supported by immunohistochemical analysis of human kidney sections, obtained from patients suffering from prolonged hyperglycaemia, due to diabetes mellitus type 2.

## Results

### Human diabetic patients and hyperglycaemic zebrafish illustrate no change in ELMO1 expression in the kidney

**Figure 1:**
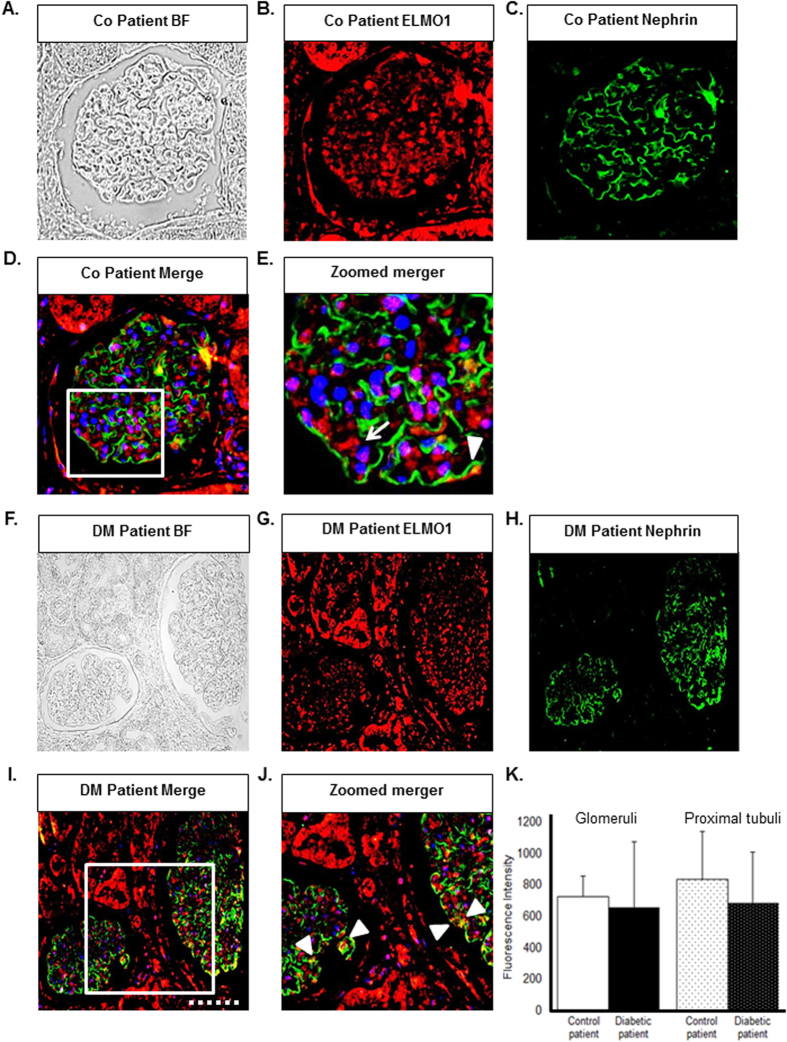
Human glomerular expression of ELMO1 is seen specifically within podocytes and remains unaltered in diabetic patients as compared to nondiabetic patients. ELMO1 expression within the glomerulus appears to be present specifically in podocytes in human control and diabetic patients. (**A**–**E**) Illustrate a glomerulus from a control patient. (**B**) Indicates ELMO1 expression, highly present in the tubules and to lesser extent within the glomerulus. (**C**) Illustrates nephrin (podocyte marker) expression in green. A merger of panels, (**D**) displays that ELMO1 expression within the glomerulus partially co-localises with nephrin, indicating that ELMO1 expression is of podocyte origin, within the renal structure. This is further emphasised in panel (**E**) where ELMO1 expression specifically in the glomerulus is indicated with white arrow head. Cytosolic ELMO1 expression within podocytes near the nuclei (blue), surrounded by extracellular nephrin staining is indicated in (**E**) with a white arrow. Similarly, (**F**–**J**) illustrate a glomerulus from a type 2 diabetic patient, displaying similar ELMO1 and nephrin expression patterns. The white dotted line in (**I**) illustrates a scale bar of 50 μm. (**K**) Illustrates quantified ELMO1 expression within renal glomerulus and the proximal tubules, in diabetic and nondiabetic patients (eight different glomeruli and proximal tubules were analysed over 5 patients per group). The graphs indicate no significant difference in ELMO1 expression under diabetic conditions as compared to the control patients (n = 5).

ELMO1 expression was analysed for potential changes within human kidney sections received from nondiabetic, diabetic (type 2) and polycystic kidney disease patients (Fig. 1 and Supplementary Figure 1). Glomeruli in nondiabetic kidneys also showed ELMO1 positive reactivity in podocytes (Fig. 1D,E), but expression was almost absent in glomerular endothelial cells (Supplementary Figure 2D,E). It was also observed, that under nondiabetic conditions, ELMO1 was also expressed within the tubules. Remarkably, ELMO1 expression remained unchanged within the kidney of patients suffering from type 2 diabetes, although the renal expressional pattern of ELMO1 was similar to that of nondiabetic patients (Fig. 1I–K; Supplementary Figures 2I,J). Unaltered expression was also observed in patients suffering from polycystic kidney disease (Supplementary Figure 1C). Thus, histological results indicate that ELMO1 is equally expressed in different kidney diseases and not differentially regulated, due to a specific pathological condition.

**Figure 2:**
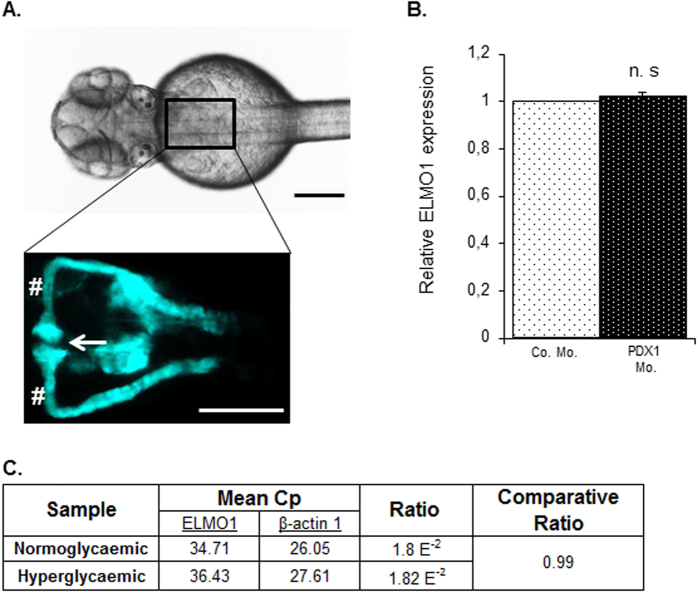
Expression of ELMO1 is observed within the zebrafish pronephros but remains unaltered under hyperglycaemic conditions as compared to normoglycaemic fish. (**A**) Illustrates the position of the pronephros in a *Tg(wt1b:EGFP*) embryo (48 hpf), visible from the dorsal view. The renal structure is composed of two tubules that are fused to form a single glomerulus (white arrow) via a neck (white hashtag). Both the scale bars represent 200 μm. (**B**) No change in relative ELMO1 expression was observed within the zebrafish pronephros under hyperglycaemic conditions (4 ng morpholino injection against insulin promotor factor 1, PDX1). Co. Mo. indicates 4 ng control morpholino. Pronephric cells were isolated for mRNA extraction via FACS sorting from *Tg(wt1b:EGFP*) embryos for cDNA synthesis and subsequent real-time qPCR analysis. (**C**) tabulates the mean Cp (crossing point of the amplifying fluorescence exceeding the background fluorescence) values of ELMO1 (target) and β-actin 1 (reference) expression.

To induce hyperglycaemia in zebrafish embryos, the morpholino technology was employed to knockdown PDX1 transiently^16^. Subsequently, we collected pronephric cells from the *Tg(wt1b:EGFP*) zebrafish line (Fig. 2A) subjected to hyperglycaemia and performed a real-time quantitative PCR, to assess expressional changes of ELMO1 within the renal system of the zebrafish embryo under normo- and hyperglycaemic conditions. It is important to note that only podocytes are labelled with EGFP within the glomerulus in the *Tg(wt1b:EGFP*) zebrafish embryos^18^. No significant change in ELMO1 expression was observed (Fig. 2B,C) within the pronephros of hyperglycaemic embryos as compared to the control. These findings indicate that the data on the zebrafish embryos is relatable to patient data, where ELMO1 expression remains unaltered in diabetic patients (Fig. 1).

### Hyperglycaemia and ELMO1 CRISPR injection lead to adverse changes in the zebrafish pronephros

**Figure 3:**
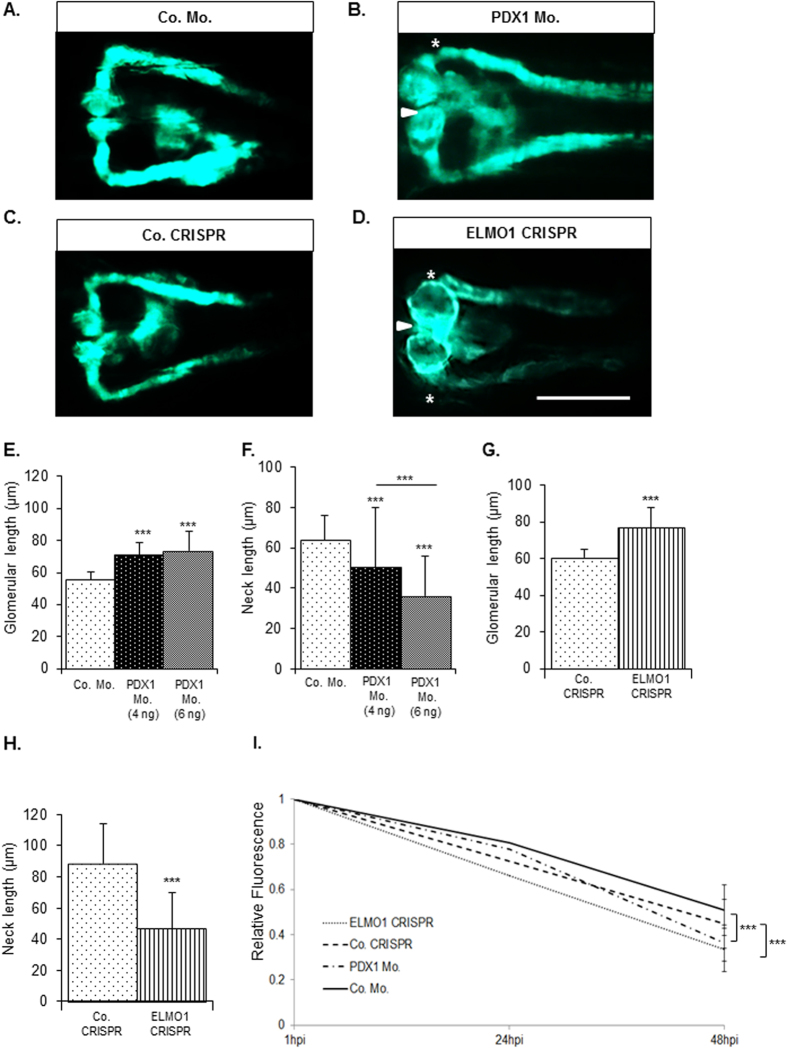
Zebrafish pronephric structure and function are adversely affected by hyperglycaemia and upon CRISPR mediated ELMO1 knockout. As compared to the zebrafish pronephros at 48 hpf in control morphants, Co. Mo., (**A**) hyperglycaemic PDX1 morphants, PDX1 Mo., (**B**) display an enlarged glomerulus (white arrow head) and highly shortened pronephric neck (white asterix). (**C**) Control crispanst (Co. CRISPR) display a normal glomerulus. (**D**) In contrast, ELMO1 crispants, (ELMO1 CRISPR), show an enlarged glomerulus (white arrow head) and a highly shortened pronephric neck (white asterix). (**E**–**H**) The altered structure in morphants and crispants has been quantified in n = 40 embryos for each condition. (**I**) Displays the quantified elevated loss of fluorescence in ELMO1 crispants (n = 38) and PDX1 morphants (n = 26) as compared to their respective controls (Co. CRISPR, n = 39 and Co. Mo., n = 30). The white line in image (**D**) represents the scale bar corresponding to 200 μm.

We next aimed to assess the role of ELMO1 in renal structural and functional development, under hyperglycaemic conditions. Consequently, effects of PDX1 knockdown and knockout of ELMO1 were analysed with respect to the zebrafish pronephros (Fig. 3). As compared to the control (Fig. 3A), the PDX1 morphants, at 48 hpf, exhibited an enlarged glomerulus, where the width was significantly increased from 60 μm in the controls to 75 μm in PDX1 morphants (Fig. 3B,E). The neck of the PDX1 morphants was greatly shortened to 50 μm in 4 ng injected PDX1 morphants and 37 μm in 6 ng injected PDX1 morphants, as compared to a length of 63 μm in the controls (Fig. 3B,F).

CRISPR/Cas9 mediated ELMO1 knockout (Supplementary Figure 3) was carried out and embryos aged 48 hpf were studied for renal phenotypes. Similar to hyperglycaemia induced changes, ELMO1 crispants exhibited alterations in the zebrafish pronephric structure. As compared to the control (Fig. 3C), ELMO1 crispants displayed a significant increase in width (from 60 μm in controls to 76 μm in the ELMO1 crispants) and strong reduction of the neck (from 80 μm in controls to 40 μm in the ELMO1 crispants) (Fig. 3D,G,H). To evaluate hyperglycaemia and ELMO1 knockout effects on renal functionality, we assessed pronephric ultrafiltration (Supplementary Figure 4). The assay to determine changes in the functionality of the zebrafish pronephros has already been established^19^. Upon heart injection of the 70 kDa Texas-Red^®^ dextran, a significantly increased loss of heart fluorescence over time, in PDX1 morphants and the ELMO1 crispants, was observed as compared to their controls (Fig. 3I). This indicates an adversity in the ultrafiltration, in both the hyperglycaemic fish and ELMO1 crispants. Thus, hyperglycaemic conditions and ELMO1 knockout, not only alter pronephric structure, but also adversely affect the function of the renal system, within zebrafish embryos.

### Hyperglycaemia induced pronephric structural and functional alterations within zebrafish embryos can be significantly rescued via ELMO1 overexpression

**Figure 4:**
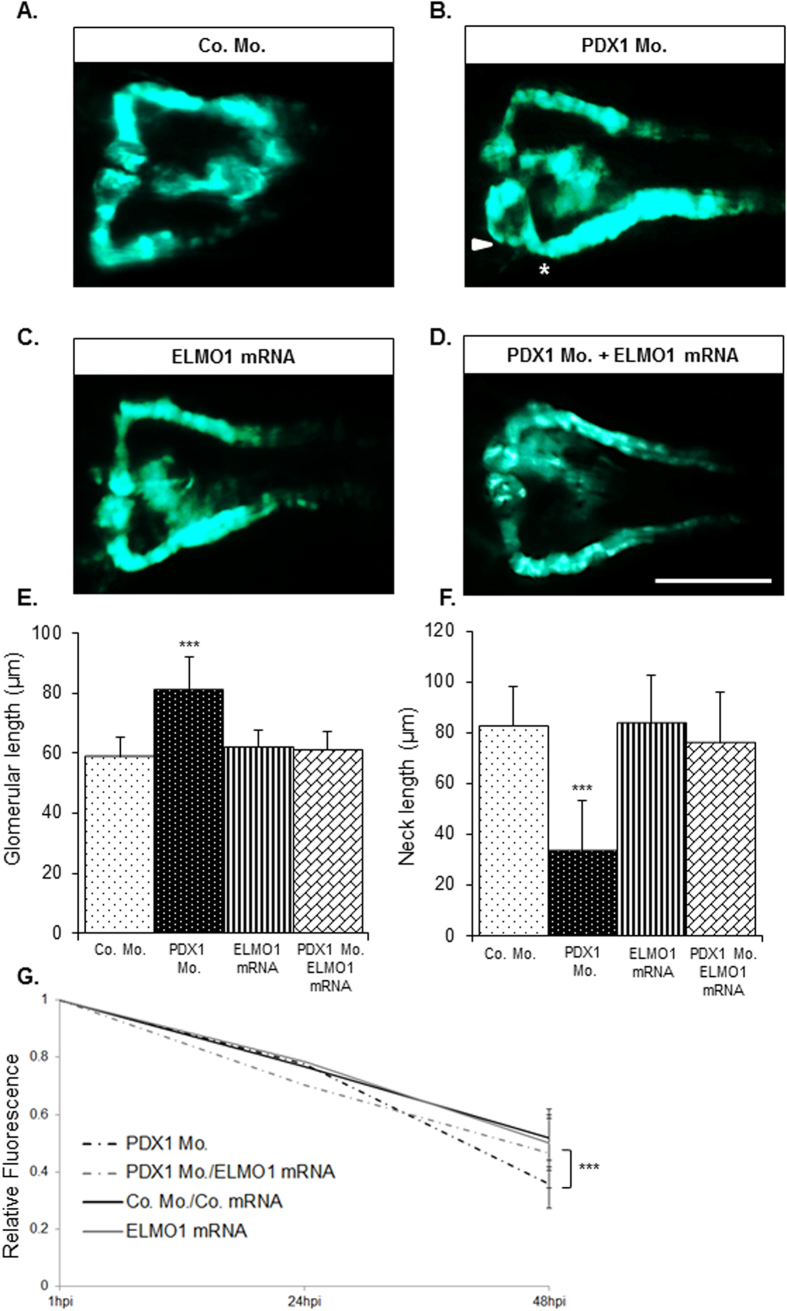
Over-expression of ELMO1 rescues the renal structural and functional phenotype caused due to hyperglycaemia. As compared to the zebrafish pronephros at 48 hpf in control morphants, Co. Mo., (**A**) hyperglycaemic PDX1 morphants, PDX1 Mo. (4 ng), (**B)** display an enlarged glomerulus (white arrow head) and a highly shortened pronephric neck (white asterix). **(C)** Overexpression of ELMO1 has no detrimental effects on the zebrafish pronephros, however, (**D)** overexpression of ELMO1 in PDX1 morphants significantly rescues pronephric phenotype. (**E**,**F)** The altered structure in PDX1 morphants and their subsequent rescue with ELMO1 overexpression is quantified in n = 40 embryos, for each condition. (**G)** Displays the quantified elevated loss of fluorescence in PDX1 morphants (n = 23) and the significant increase in fluorescence within hyperglycaemic fish upon ELMO1 overexpression (Double Co. Mo. and mRNA, n = 23; ELMO1 mRNA, n = 23; and PDX1 Mo. with ELMO1 mRNA, n = 23).

As hyperglycaemia and knockout of ELMO1 have similar detrimental effects on the zebrafish pronephric structure and function, we aimed to establish whether overexpression of ELMO1, could rescue hyperglycaemic renal alterations as shown in Fig. 3. Overexpression of ELMO1^13^,^14^ (Supplementary Figure 6) could beneficially restore the compromised pronephric structure within PDX1 morphants (Fig. 4A–D). The structural changes represented by glomerular enlargement and reduced pronephric neck length in PDX1 morphants (Fig. 4B,E,F) were almost entirely normalized upon the co-injection of ELMO1 mRNA. Similarly, increased clearance of the 70 kDa Texas-Red^®^ dextran within PDX1 morphants was decreased to levels corresponding to controls, upon ELMO1 overexpression (Fig. 4G). Thus, under hyperglycaemic conditions, overexpression of ELMO1 was found to rescue the altered renal structure and function within zebrafish embryos.

### Podocyte foot processes are detrimentally affected in PDX1 morphants and ELMO1 crispants, altering the glomerular filtration barrier

**Figure 5:**
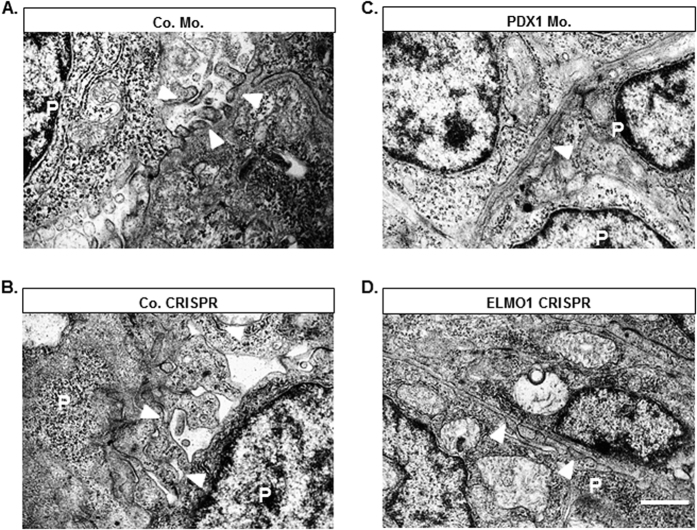
Knockdown of PDX1 and knockout of ELMO1 causes alterations in podocytic structure within zebrafish embryos aged 48 hpf. (**A,B**) Podocytes (P) in Co. Mo. and Co. CRISPR zebrafish embryos aged 48 hpf are well developed and have well defined foot processes that interdigitate amongst themselves to form functional filtration barriers (white arrow heads). (**C**) Upon PDX1 knockdown, complete distrophy of podocyte foot processes is observed. (**D**) Upon ELMO1 knockout, foot processes are immature and are unable to interdigitate to form an appropriate filteration barrier. The white line indicates the scale bar corresponding to 1 μm length. Number of embryos analysed per sample group were 10.

Since the renal filtration barrier is drastically altered in a diabetic nephropathy setting^4^, we aimed to determine if at an electron microscopic level, PDX1 morphants and ELMO1 crispants were affected, especially with respect to podocyte structure and the formation of the ultrafiltration barrier. Consequently, we found that PDX1 morphants (48 hpf) display a highly dystrophic filtration barrier as compared to their controls (Fig. 5A,C), where the podocyte structure contributed to a highly under-developed filtration barrier. Foot processes between adjacent podocytes did not interdigitate appropriately and urinary space was absent. ELMO1 crispants (48 hpf) also exhibited inappropriate podocyte development and filtration barrier, as compared to the control (Fig. 5B,D). Specifically, in ELMO1 crispants foot processes did not develop and interdigitate to form the filteration barrier and podocytes appeared unable to filter the blood plasma (Fig. 5D). Thus, it is further established that, even at a subcellular level, hyperglycaemia and ELMO1 CRISPR injections lead to podocyte structural adversities.

### Pancaspase inhibitor, zVAD-fmk, treatment can restore altered structural and functional changes in ELMO1 CRISPR injected embryos

**Figure 6:**
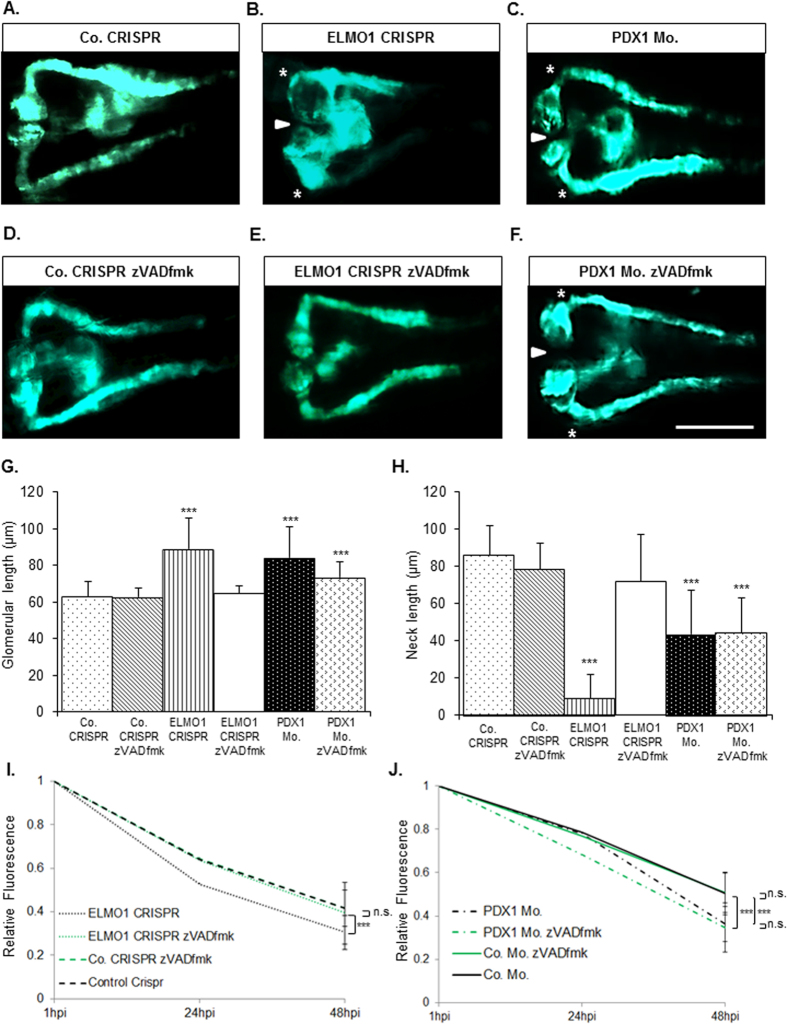
Compromised pronephric structure and ultrafiltration in ELMO1 crispants, but not in PDX1 morphants, can be beneficially reversed upon treatment with pancaspase inhibitor, zVAD-fmk. Upon treatment with zVAD-fmk (300 μM), pronephric structural defects in ELMO1 crispants at 48 hpf, are significantly restored to normalcy (**A**,**B**,**D**,**E**); n = 47 for each condition). The pancaspase inhibitor causes no adversity in the control embryos at 48 hpf (**D**). However, no significant restoration in pronephric structure is observed with zVAD-fmk treatment of PDX1 morphants, at the same age (**C,F**); n = 47 for both conditions). The white line in image (**F**) represents the scale bar corresponding to 200 μm. The significant restoration of the glomerular width and neck via zVAD-fmk treatment in ELMO1 crispants as well as the lack of rescue in PDX1 morphants are quantified in graphs (**G**,**H**). Altered ultrafiltration in ELMO1 crispants is also improved significantly upon treatment with zVAD-fmk as seen in graph (**I**) but not in PDX1 morphants, as observed in (**J**) The n values for each sample set used for the functional assay are as follows: Co. CRISPR (22), Co. CRISPR with zVAD-fmk (23), ELMO1 CRISPR (22), ELMO1 CRISPR with zVAD-fmk (17), Co. Mo. (23), Co. Mo. with zVAD-fmk (23), PDX1 Mo. (26) and PDX1 Mo. with zVAD-fmk (22).

Recently, ELMO1 has been established to protect endothelial cells from apoptosis during angiogenesis^14^. It was hypothesized that a similar function is being performed by the protein in the renal system. zVAD-fmk has been previously utilized as an apoptotic inhibitory compound on zebrafish embryos^20^. Anti-apoptotic treatment of zebrafish embryos was carried out at the age of 24 hpf for 24 hours, in order to observe any changes in the pronephros (Fig. 6). It was observed that upon treatment with zVAD-fmk, ELMO1 crispants showed a significant rescue of pronephric structure as compared to those embryos without treatment (Fig. 6A,B,D,E,G,H). The zVAD-fmk treatment reduced significantly the width of the enlarged glomerulus from 81 μm to 60 μm in length, and restored the length of the pronephric neck of the compromised ELMO1 embryos from 18 μm to 75 μm in length, which are comparable to the control embryos (Fig. 6G,H). Ultrafiltration was also analysed in the ELMO1 crispants upon zVAD-fmk treatment. zVAD-fmk treatment of the ELMO1 CRISPR injected embryos also considerably rescued the fluorescence loss from the heart as compared to those ELMO1 crispants without treatment (Fig. 6I). Interestingly, it was found that the pronephros in PDX1 morphants displayed no signs of improvement in structural and functional integrity, upon pancaspase inhibitor treatment (Fig. 6C,F,G,H,J). Thus, the loss of ELMO1 causes structural and functional phenotype within the zebrafish embryos due to an increase of apoptosis, which can be rescued upon pancaspase inhibitor treatment. However, hyperglycaemia induced pronephric changes within the zebrafish embryos cannot be rescued with zVAD-fmk treatment alone, owing to the probable activation of other detrimental mechanisms^21^.

**Figure 7:**
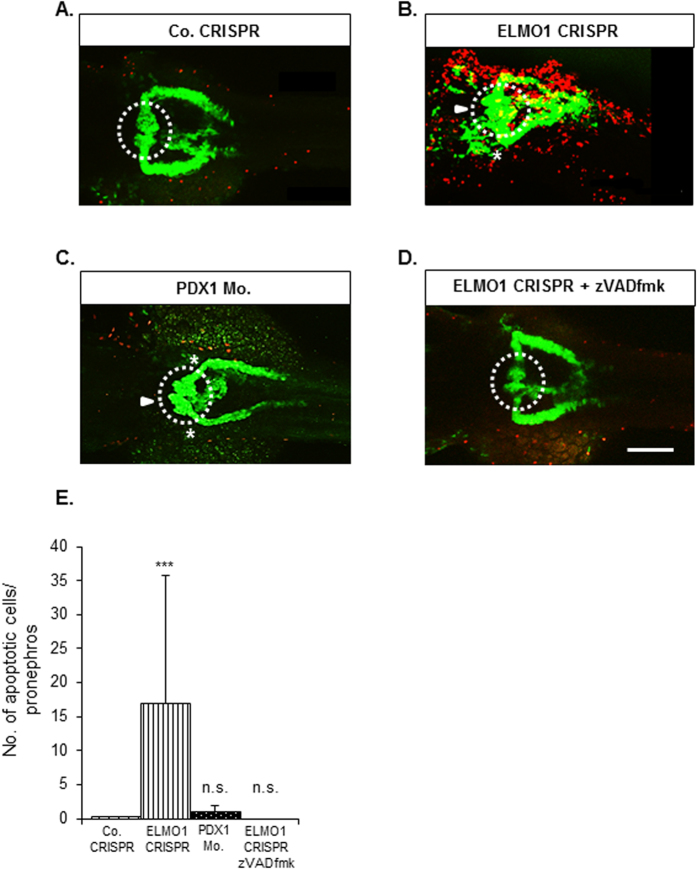
ELMO1 knockout, but not hyperglycaemia, causes an increase in apoptotic cells within the zebrafish embryo as well as the pronephros, which can be rescued with pancaspase inhibitor, zVAD-fmk. (**A**) TUNEL assay was carried out on Co. CRISPR injected *Tg(wt1b:EGFP*) embryos at 48 hpf. The embryos showed no incidence of apoptosizing cells (indicated with the red spots) within the renal structure. (**B**) ELMO1 CRISPR injected embryos showed a strong incidence of apoptosizing cells (red spots) universally as well as within the renal structure. (**C**) Indicated that hyperglycaemic embryos do not display increased apoptosis within the pronephros and (**D**) indicates that ELMO1 crispants displayed normalized renal structure and a drastic reduction of apoptosis, upon zVAD-fmk treatment, which is quantified in the graph. (**E**) The white line in (**D**) indicates a scale bar representing 100 μm, the white arrow head indicated an enlarged glomerulus and the white asterisk illustrates shortening of the glomerular neck of the ELMO1 crispants as compared to the control. The white dotted circle encloses the glomerulus of the zebrafish pronephros. The number of embryos per condition analysed are 11.

Finally, we aimed to establish if ELMO1 knockout indeed causes an increased incidence of apoptotic cells. The TUNEL assay was employed on ELMO1 crispant embryos, in order to visualize any significant increase in apoptosis universally and especially within the pronephros (Fig. 7). ELMO1 crispants displayed a significantly much higher incidence of apoptotic cells as compared to their controls, at 48 hpf (red dots, Fig. 7A,B). This increase in number of apoptotic cells is not only observed throughout the embryos, but also within the pronephros in the ELMO1 crispants as compared to their controls (Fig. 7E). This data has been further demonstrated with an activated Caspase3 assay (Supplementary Figure 7). Furthermore, zVADfmk treatment of ELMO1 crispants not only restored pronephric structural adversities but also drastically reduced the incidence of apoptotic cells within these crispants (Fig. 7D,E). Hyperglycaemia did not lead to an increase in apoptotic cells within the pronephros (Fig. 7C,E). Thus, it is evident that ELMO1 knockout in zebrafish embryos leads to an increase in apoptosis within the entire organism, and particularly within the renal system.

## Discussion

**Figure 8:**
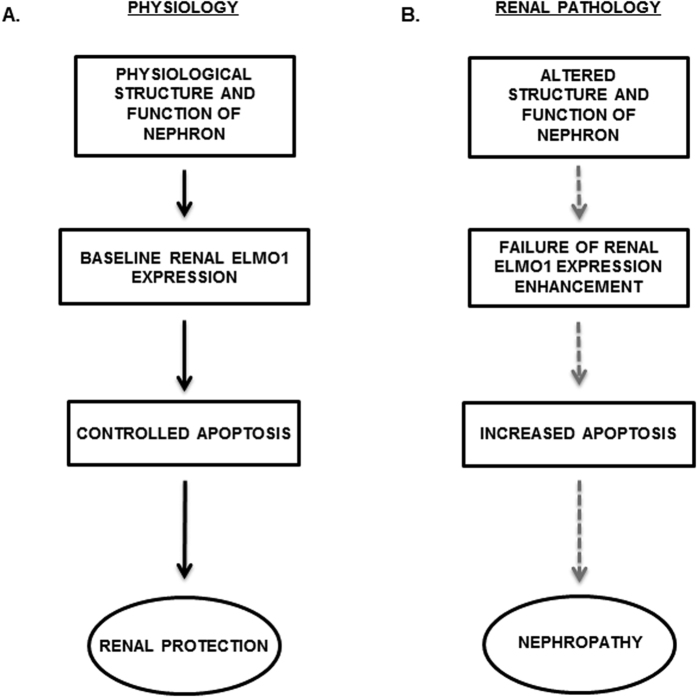
Model of the functional role played by ELMO1 in the development of nephropathy under diabetic conditions. **(A)** ELMO1 plays a protective function for the renal system by safe guarding the organ from apoptosis, under physiological conditions. (**B)** In renal pathology, failure in increased ELMO1 expression is unable to cope with the disease induced apoptosis, which eventually leads to nephropathic alterations.

In this study, we have established an important role of ELMO1 in renal function, under hyperglycaemic conditions, within the zebrafish and successfully translated our finding to the pathogenesis of human diabetic nephropathy (Fig. 8). ELMO1 not only shields endothelial cells from apoptosis^14^, but also functions to protect glomerular cells from the phenomenon, under high glucose conditions. Hyperglycaemic conditions in zebrafish embryos caused by PDX1 knockdown^16^, do not induce ELMO1 expression within the pronephros. This coincides with our data from type 2 diabetic patients, where no change in ELMO1 expression was observed within the kidney, compared to nondiabetic and polycystic kidneys, respectively. Thus, our study suggests that the work performed on zebrafish embryos, can be translated to a clinical setting, where ELMO1 could protect the diseased kidney from increased apoptosis, irrespective of the underlying renal disease. Overexpression of ELMO1 within mammalian (rat) glomeruli has been previously linked to extracellular matrix protein accumulation under diabetic conditions, which is predicted to be a detrimental cause, leading to the pathogenesis of diabetic nephropathy^11^. However, several GWAS studies have indicated SNPs within the *elmo1* gene relate to the susceptibility of patients suffering from diabetes to develop nephropathy, in various human populations^8^,^9^,^10^. These studies thus strongly indicate that mutations within the *elmo1* gene may lead to pathogenesis of DN, thereby suggesting a protective function of this protein concerning the disorder. In accordance with the GWAS indication, we have established that overexpression of ELMO1 actually helps to protect renal structure from pathogenesis under hyperglycaemia. PDX1 knockdown causes similar detrimental alterations within the pronephric structure and function in zebrafish embryos, as observed within the pronephros of ELMO1 crispants. Furthermore, adversities within the renal structure of the zebrafish PDX1 morphants are significantly restored upon simultaneous overexpression of ELMO1. These findings lead to the conclusion that the increase in ELMO1 expression would be a protective response of the pronephros to try and compensate against the harmful effects of the potentially damaging hyperglycaemia. To additionally strengthen this inference, ELMO1 knockout was found to be damaging to the zebrafish pronephric structure and function.

It is known previously that podocyte-specific deletion of Rac1 in mice, a protein activated by ELMO1 and Dock180 interaction, causes foot process effacement of these cells; but only leads to loss of podocytes under hyperglycaemic conditions^22^. Since ELMO1, the upstream regulator of Rac1, is associated with endothelial cell protection against apoptosis and with pathogenesis of DN, we predicted that ELMO1 plays the same survival role in the renal system subjected to physiological and hyperglycaemic conditions. Further investigation of ELMO1 crispants displayed a significantly higher incidence of apoptotic cells within the whole embryo and especially within the pronephric structure, which we suggested to be the cause of structural and functional renal adversities. To confirm this hypothesis, treatment of ELMO1 crispants with the pancaspase inhibitor, zVAD-fmk, showed restoration of the renal disruptions, both structural and functional. Therefore, it is evident that ELMO1 protects renal cells from increased apoptosis within zebrafish embryos. Thus, we expect that an upregulation of ELMO1 in the diabetic patients would likely protect the nephron from increased apoptosis, ultimately delaying the onset of diabetic nephropathy.

To ensure the relevance of our research, it was essential to transfer our findings in the zebrafish embryos to human patient data. Hence, kidney sections from nondiabetic, diabetic and polycystic kidney disease patients were tested for ELMO1 expression. We found that these patients, that we analysed, displayed an unaltered expression of ELMO1 within the renal parenchyma. This is in accordance with our research conducted on zebrafish embryos, but is in contrast to a recent published study which suggested that low ELMO1 expression prevents diabetic nephropathy development in mice^23^. It is common knowledge that no animal model completely adheres to the pathogenic mechanisms of human diabetic nephropathy. For example, mice with different background strains have variable susceptibility to developing diabetic nephropathy. Additionally, all criteria for diabetic nephropathy such as renal histolopathology and abnormal glomerular filtration rate are not completely relatable to mice models of diabetic nephropathy^24^,^25^. Thus, keeping this discrepancy of translatable murine data to human diabetic nephropathy in mind, a need for a new animal model is evident. Our data reinstates the relevance of zebrafish as a versatile animal model in basic diabetes research^26^. The zebrafish is known for its ease to work with, low maintenance costs and high throughput. Our study further displays that the zebrafish is a valuable model not only to test an implicated protein in diabetic nephropathy but also to understand the underlying mechanisms of the protein within the renal system; establish possible treatments, and translate these finding to the human setting.

In conclusion, upregulation of ELMO1 is advantageous for renal cells in zebrafish, especially under unfavourable hyperglycaemic conditions, which has been clearly established in the present study. Future prospects should thus focus on the induction of ELMO1 in diabetic conditions, in order to compensate for unfavorable effects of hyperglycaemia on the renal structure, contributing to diabetic nephropathy. Our findings clearly show that ELMO1 protects the renal system under hyperglycaemic conditions, via the reduction of apoptosis, within zebrafish embryos. The research is further translated in human patients suffering from long term hyperglycaemia leading to development of nephropathy.

## Methods

### Research design and methods

#### Zebrafish lines and husbandry

All experimental procedures on animals were approved by the local government authority, Regierungspräsidium Karlsruhe (license no.: 35-9185.81/G-98/15) and carried out in accordance with the approved guidelines. Embryos of the lines *tg(fli1:EGFP*)^27^ and *Tg(wt1b:EGFP*)^18^ were raised and staged as previously described^28^. Embryos were kept in E3 medium (5 mM NaCl, 0.17 mM KCl, 0.33 mM CaCl_2_, 5–10% methylene blue) at 28.5 °C with 0.003% 1-phenyl-2-thiourea (Sigma) to suppress pigmentation.

#### Inhibitors, antibodies and reagents

Goat anti-actin (1:1000) (Santa Cruz Biotechnology), goat anti- ELMO1 (1:200) (Everest Biotechnology) were used for Western blotting. Immunohistochemistry (IHC) was done with a polyclonal rabbit anti-ELMO1 antibody (1:20), and rabbit anti-podocin antibody (1:400) (Sigma-Aldrich). CONFIRM anti-CD34 (QBEnd/10) Primary Antibody (mouse, 1:25) was used as an endothelial marker in IHC stainings (Ventana Medical Systems). Human nephrin antibody (Sheep polyclonal; R&D systems) (1:20) and, human CD34 (mouse monoclonal; ThermoFischer) (1:40) were used to stain human kindey sections for immune-fluoro-histochemistry. Secondary antibodies (Molecular Probes; Life Technologies) used were as follows: Alexa Fluor 546 goat anti-rabbit (for ELMO1 detection; 1:400); Alexa Fluor 488 donkey anti-sheep (for nephrin detection; 1:400), and Alexa Fluor 488 goat anti-mouse (for CD34 detection; 1:400). Rabbit anti-activated Caspase 3 antibody was used at a dilution of 1:500 (Fisher Scientific). Texas-Red^®^ tagged 70 kDa dextran (Molecular Probes) was used for renal functional assays^19^. Proteinase K (10 mg/ml stock) was utilized for embryo digestion and linearized plasmid treatment (Roche Recombinant PCR grade). Zebrafish embryos were subjected to 300 μM of the pancaspase inhibitor, zVAD-fmk (Sigma-Aldrich) incubation at 24 hpf for 24 hours.

#### CRISPR control, ELMO1 gRNA and Cas9 mRNA synthesis

ELMO1 gRNA: ELMO1-CRISPR-for#1: TAGGTGGCCATCGAGTGGCCTG (underlined region is the gRNA target site) and ELMO1-CRISPR-rev#1: AAACCAGGCCACTCGATGGCCACC, oligos were cloned into the plasmid, pUC19 (Addgene)^29^. BamHI-HF (Biolabs) was used for pUC19-linearisation. Cas9 mRNA was synthesised from pT3TS^30^ [Addgene] after linearising with XbaI (Biolabs). Plasmids were purified with the PCR purification kit (Qiagen). *In vitro* transcription was done by T7 MEGAshortscript kit (Invitrogen) (control and ELMO1 gRNA) and mMESSAGE MACHINE Kit (Invitrogen) (Cas9 mRNA). Purification of RNA after TURBO DNAse treatment was done via the MiRNeasy Mini (Qiagen) and RNeasy Mini (Qiagen) kits.

#### Morpholinos and mRNA

Morpholino oligonucleotides used were: SB-PDX1-Mo: 5′-GATAGTAATGCTCTTCCCGATTCAT-3′ (targets the zebrafish PDX1 translation start site), and control-MO: 5′-CCTCTTACCTCAGTTACAATTTATA-3′^31^. Synthesis and injection of zebrafish ELMO1 and control mRNA were performed using the SP6 mMessage mMachine Kit (Ambion) as previously described^13^.

#### Injections of morpholinos, mRNA and gRNA

Morpholinos were diluted to 4 or 6 μg/μl in 0.1 M KCl. mRNA was diluted to 300 pg/nl; ELMO1 gRNA and Cas9 mRNA were diluted to 100 pg/nl and 150 pg/nl respectively, in 0.1 M KCl. One nanoliter of morpholino or mRNA was injected into the embryos at the 1-cell stage.

#### Western blot analysis

Zebrafish embryos were collected at 48 hpf for Western blot analysis. Western blot was performed as described before^32^.

#### FACS sorting

48 hpf Embryos of the *Tg(wt1b:EGFP*) were incubated in calcium free ringer solution (116 mMNaCl, 2.9 mM KCl and 5 mM HEPES, pH 7.2), for 15 min. The embryos were then deyolked by rapid pipetting, using a Gilson P200, washed with PBS, centrifugated (5 min, 1400 rpm). Embryos were digested for 20 min with 0.25% Trypsin in 1 mM EDTA (GIBCO), stopped with a stop solution (1x PBS, 10% FCS and 2 mM CaCl_2_). Embryos were resuspended in a FACS sorting buffer and passed through a 30 μm filter (Partec Cell Trics). FACS analysis was performed by the Mannheim Cell Sorting Core Facility of the Medical Faculty, using a BD FACSAria. The GFP positive and negative cells were obtained in PBS, spun down and resuspended in RLT-Buffer with β-mercapthoethanol (Carl Roth GmbH). mRNA extraction was performed using the RNeasy Mini Kit (Qiagen). cDNA synthesis was carried out using the SuperScript^TM^- First Strand Kit (Invitrogen).

#### Real-time quantitative PCR

Primer design for ELMO1 (target) and β-actin 1 (reference) for zebrafish was done by Roche Universal ProbeLibrary Assay Design Center. ELMO1: forward primer-5′-‘gcaccatcaggatgcttaca-3′; reverse primer- 5′-‘cggacactcatgtttatcctca’-3′ and that for β-actin 1: forward primer- 5′-‘acggtcaggtcatcaccatc’-3′ and reverse primer- 5′-‘tggataccgcaagattccat’-3′. Probes #80 and #11 (Roche UPL) were used for ELMO1 and β-actin 1, respectively. 2x sentiFAST probe No-ROX mix (Bioline) was used in each 96-well reaction plate (Axon). A Roche light cycler^®^ 480 was used for the qPCR. Comparative ratio for ELMO1 expression was calculated with the 2^-ddCP^ 33. The real-time quantitative PCR was repeated twice with a total of 6 replicates for each condition. The number of embryos used for the hyperglycaemic condition was 1017 and 895 for normoglycaemic conditions. The comparative ratio (Pfaffl formula) indicates no significant difference between ELMO1 expression levels.

#### Ultrafiltration assay

72 hours post-fertilisation, 5 nl of Texas-Red^®^ tagged 70 kDa dextran (2 ng/ml in PBS) was injected into the heart of *tg(fli1:EGFP*) embryos. Sequential images of the living fish were taken 1, 24 and 48 hours post injection (hpi) using an inverted microscope (Leica DMI 6000B) with a camera (Leica DFC420 C) and the Leica LAS application suite 3.8 software. Maximum fluorescence intensity in the heart area was measured using NIH’s ImageJ application (Supplementary Figure 3). The fluorescence values were compared in relative units of brightness for each fish.

#### Electron Microscopy (EM) study

*Tg(wt1b:EGFP*) embryos were injected at the 1-cell stage with control and PDX1 morpholinos (6 ng), control CRISPR gRNA/Cas9 mRNA (100 + 150 pg) and ELMO1 CRISPR gRNA/Cas9 mRNA (100 + 150 pg). Embryos were fixed at 48 hpf in 0.01 Na-cacodylate buffer pH 7.4 for 24 h at 4 °C and sent for EM analysis.

#### Microscopy and analysis of pronephric alteration

For *in vivo* imaging *Tg(wt1b:EGFP*) embryos were embedded in 1% low melting point agarose (Promega), dissolved in E3 medium. Images were taken with a Leica DFC420 C camera, attached to a Leica MZ10 F modular stereo microscope. Quantification of the pronephron was done using the Leica LAS V4.8 software. Imaging was carried out for TUNEL stained *Tg(wt1b:EGFP*) via a DMIRE2 microscope with Leica TCS SP2 True Confocal Scanner and a DM6000 B microscope with Leica TCS SP5 DS scanner. Images were taken with 600 Hz, 1024 × 512 pixels and z-stacks were 1 μm thick.

#### TUNEL assay

TUNEL assay was performed on 48 hpf *Tg(wt1b:EGFP*) embryos, using the ApopTag^®^ Red *In Situ* Apoptosis Detection Kit (Millipore). The embryos were deyolked and fixed over-night at 4 °C, in freshly prepared 4% PFA/PBS. After washing in PBST (1x PBS/0.1% Tween-20), sequential dehydration with increasing concentrations of methanol, was done to store them in 100% methanol overnight at −20 °C. Embryos were rehydrated and treated with Proteinase K (10 μg/ml, Roche) for 21 min. TUNEL protocol was hence followed as previously described^14^.

#### Activated Caspase 3 assay

48 hpf aged *Tg(wt1b:EGFP*) embryos were deyolked and fixed over-night at 4 °C, in freshly prepared 4% PFA/PBS. After washing in PBST (1x PBS/0.1% Tween-20), sequential dehydration with increasing concentrations of methanol, was done to store them in 100% methanol overnight at −20 °C. Embryos were rehydrated and washed (3x) in PBST for 20 min. After blocking for 1 hour at RT (Block buffer: 1x PBST, 10% heat-inactivated fetal bovine serum, 2% bovine serum albumin), embryos were subject to an over-night incubation with rabbit anti-activated caspase 3 antibody (1:500) at 4 °C. After sequential washing with PBST and blocking for another hour, embryos were incubated with Alexa Fluor 546 goat anti-rabbit (1:400), over-night at 4 °C. After washing 3x with PBST for 20 mins, embryos were thus prepared for examination under a confocal microscope.

#### Immunohistochemistry (IHC) and fluoro-IHC for ELMO1

Human kidney tissue samples were provided by the tissue bank of the National Center for Tumor Diseases (NCT, Heidelberg, Germany) in accordance with the regulations of the tissue bank and the approval of the ethics committee of Heidelberg University.

For IHC analysis 5 non-diabetic (2/3 male/female patients aged 39–71 years), diabetic (2/3 male/female patients aged 34–76 years) and polycystic kidneys (4/1 male/female patients aged 54–67 years) were analysed. All kidney organs were removed during organ recession between 2007–2015 for all patients. All diabetic patients suffered from long term type 2 diabetes and developed nephropathy where kidney function was reportedly disrupted. Two diabetic patients showed other signs of diabetic complications such as retinopathy. All diabetic patients are assured to be insulin dependent. Sections of formalin-fixed, paraffin-embedded (FFPE) kidneys, provided by the NCT Tissue Bank, were cut at a thickness of 1 μm (HM 340E Electronic Rotary Microtome, Thermo Scientific), dryed and stored at room temperature. IHC analysis was performed by using BenchMark ULTRA (Ventana Medical Systems). Antigens were retrieved with Protease 1 (12 min, room temperature, Ventana Medical Systems) and ELMO1 was detected with a polyclonal rabbit anti-ELMO1 antibody at 36 °C for 24 minutes, using the OptiView DAB IHC Detection Kit (Ventana Medical Systems). Podocin and CD34 were detected with their respective antibodies at 36 °C for 24 minutes, using the ultraView Universal Alkaline Phosphatase Red Detection Kit (Roche). NanoZoomer slide scanner (Hamamatsu) visualised tumor-free kidney parenchym and imaged with the Aperio ImageScope viewing software (Leica Biosystems, version 11.0.2.725).

For immunofluorescence staining, citrate buffer antigen revival method was used after de-paraffination was performed on kidney sections. After which, sections were blocked for an hour and incubated overnight at 4 °C with primary and then secondary antibodies. Fluorescent images were taken using a Zeiss Axio Imager D1 with a Axiocam mRm camera.

#### Quantification and statistics

TUNEL assay confocal images were processed using NIH’s ImageJ application. Apoptotic cell number/area was determined by the analyze particles tool of ImageJ, within the entire pronephric structure. Alterations in the pronephric structure were measured and quantified by measuring size in μm of glomerular width and neck. Immunofluorescence staining analysis for ELMO1 expression in human kidney sections was carried out by measuring integrated density of the fluorescence value representing ELMO1 protein expression, via NIH’s ImageJ application. Background fluorescence was duly substracted and corresponding averages/group were compared using a student *t*-test. The number of patients analysed per group were 5 and 8 glomeruli and tubules taken from 4 different stained sections, was utilized for ELMO1 quantification. Statistical significance between different groups was analysed using Student’s *t*-test. Results are indicated as the mean value +/−SD. P-values <0.05 were considered as significant *<0.05, **<0.01, and ***<0.001.

## Additional Information

**How to cite this article**: Sharma, K. R. *et al.* ELMO1 protects renal structure and ultrafiltration in kidney development and under diabetic conditions. *Sci. Rep.* **6**, 37172; doi: 10.1038/srep37172 (2016).

**Publisher’s note**: Springer Nature remains neutral with regard to jurisdictional claims in published maps and institutional affiliations.

## Supplementary Material

Supplementary Information
