# Supplementary material for: ELMO1 protects renal structure and ultrafiltration in kidney development and under diabetic conditions
Source: Sci Rep. 2016 Nov 16;6:37172. doi: 10.1038/srep37172 (PMC5111104; doi:10.1038/srep37172)
Supplement: Supplementary Information [file srep37172-s1.pdf]

# Supplemental information

## ELMO1 protects renal structure and ultrafiltration in kidney development and under diabetic conditions

Krishna Rakesh Sharma[1#], Karl Heckler[1#], Sandra J. Stoll[1,11], Jan-Luuk Hillebrands[2], Katharina Kynast[3], Esther Herpel[3,4], Stefan Porubsky[5], Marlies Elger[6], Boris Hadaschik[7], Karen Bieback[8], Hans-Peter Hammes[9], Peter P. Nawroth[10] and Jens Kroll[1,11*]

[1]Department of Vascular Biology and Tumor Angiogenesis, Center for Biomedicine and Medical Technology Mannheim (CBTM), Medical Faculty Mannheim, Heidelberg University, Mannheim, Germany [2]Department of Pathology and Medical Biology, Division of Pathology, University Medical Center Groningen, Groningen, The Netherlands, [3]Institute of Pathology, Heidelberg University, Heidelberg, Germany, [4]Tissue Bank of the National Center for Tumor Diseases (NCT), Heidelberg, Germany, [5]Institute of Pathology, Medical Faculty Mannheim, Heidelberg University, Mannheim, Germany, [6]Institue of Neuroanatomy, Medical Faculty Mannheim, Heidelberg University, Mannheim, Germany, [7]Department of Urology, Heidelberg University Hospital, Heidelberg, Germany, [8]Institute of Transfusion Medicine and Immunology, Medical Faculty Mannheim, Heidelberg University, Mannheim, Germany, [9]Fifth Medical Department, University Medical Centre Mannheim, Mannheim, Germany, [10]Department of Medicine I and Clinical Chemistry, Heidelberg University, Heidelberg, Germany and [11]Division of Vascular Oncology and Metastasis, German Cancer Research Center (DKFZ-ZMBH Alliance), Heidelberg, Germany

Word count; ABSTRACT: 197

Running title: ELMO1 in hyperglycaemic zebrafish

#Both authors have contributed equally to this study

*Correspondence should be addressed to:

Prof. Dr. Jens Kroll
Center for Biomedicine and Medical Technology Mannheim (CBTM)
Dept. of Vascular Biology and Tumor Angiogenesis, Medical Faculty Mannheim,
Heidelberg University, Germany
Ludolf-Krehl-Str. 13-17
68167 Mannheim, Germany
Phone: +49-(0)621-383-9965
Fax: +49-(0)621-383-9961
Email: jens.kroll@medma.uni-heidelberg.de
www.angiolab.de

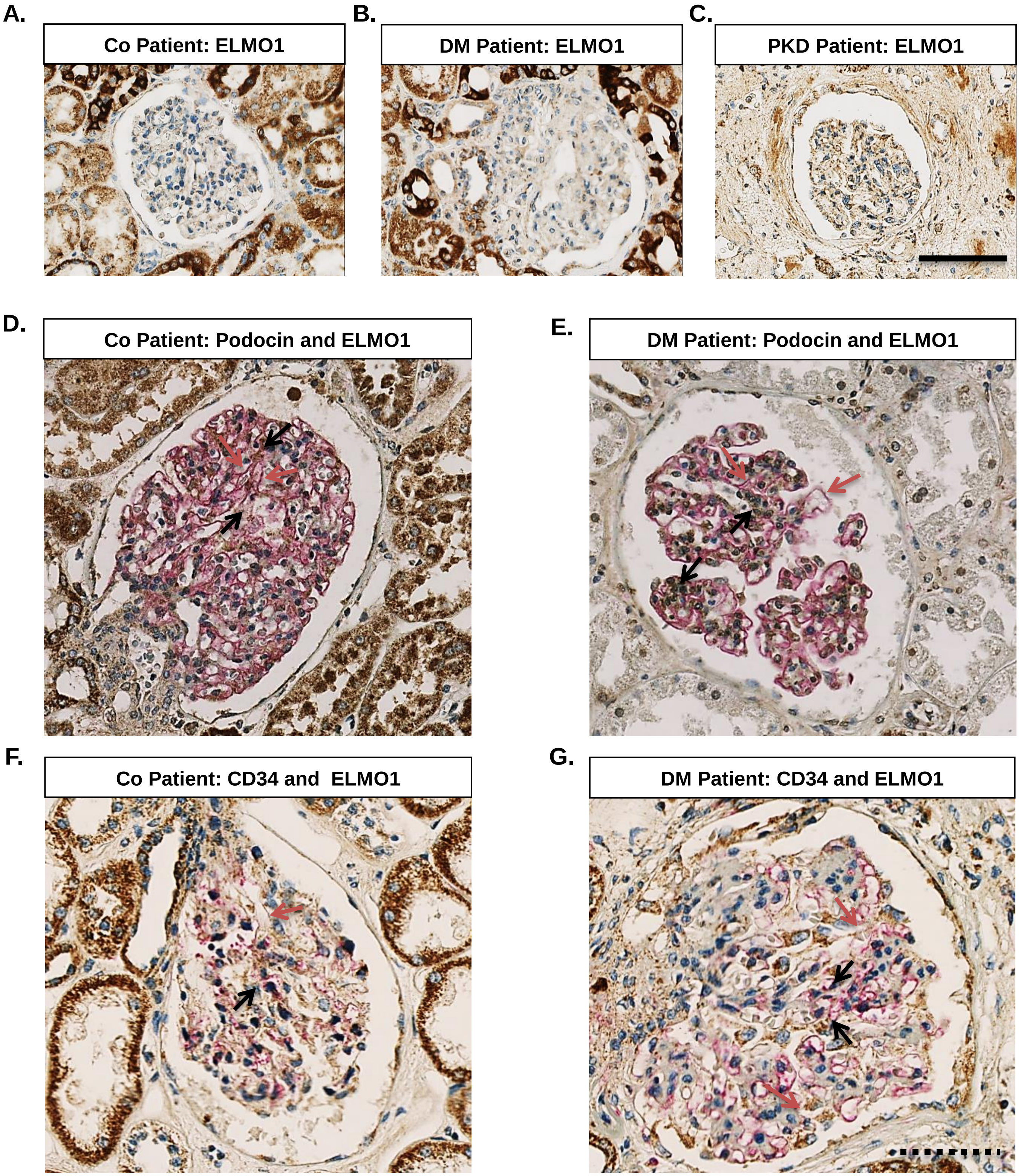




Supplementary figure 1

***Supplementary figure 1:*** **Unaltered ELMO1 expression within the glomerulus of human diabetic patients as compared to nondiabetic patient samples. A.** The kidney section taken from a nondiabetic patient displays a morphologically normal glomerulus amongst renal tubules. ELMO1 expression, illustrated by the brown staining within the tubules, but is also seen within the glomerulus. **B.** The kidney section taken from a type 2 diabetic patient displays a pathological glomerulus. ELMO1 expression within the glomerulus of type 2 diabetic patients (n=5) is not significantly altered as compared to the control in A. **C.** ELMO1 expression does not change in human patients (n=5) suffering from polycystic kidney disease, as compared to a control. However, it is appreciated that the kidney morphology within this sample is completely disrupted by fibrosis. **D., E.,** show the co-expression of ELMO1 (brown stain indicated with a black arrow) with respect to podocin (magenta stain indicated with red arrows) in the podocytes control and Type 2 diabetic patient, respectively. ELMO1 expression is observed to be contained within the cells stained with podocin on their surface. **F., G.,** show the double staining of ELMO1 (brown stain indicated with a black arrow) with respect to CD34 (magenta stain indicated with red arrows) in the glomerular endothelial cells of a control and Type 2 diabetic patient, respectively. ELMO1 expression does not seem to be contained within the cells stained with CD34 on their transmembrane, indicating lack of co-expression of ELMO1 in glomerular endothelial cells. The black line in 1C represent a scale bar of 100 µm for figures 1A., B., and C., whilst the black dotted line in 1G, represents a scale bar of 100 µm for figures 1D., 1E., 1F., and 1G respectively.

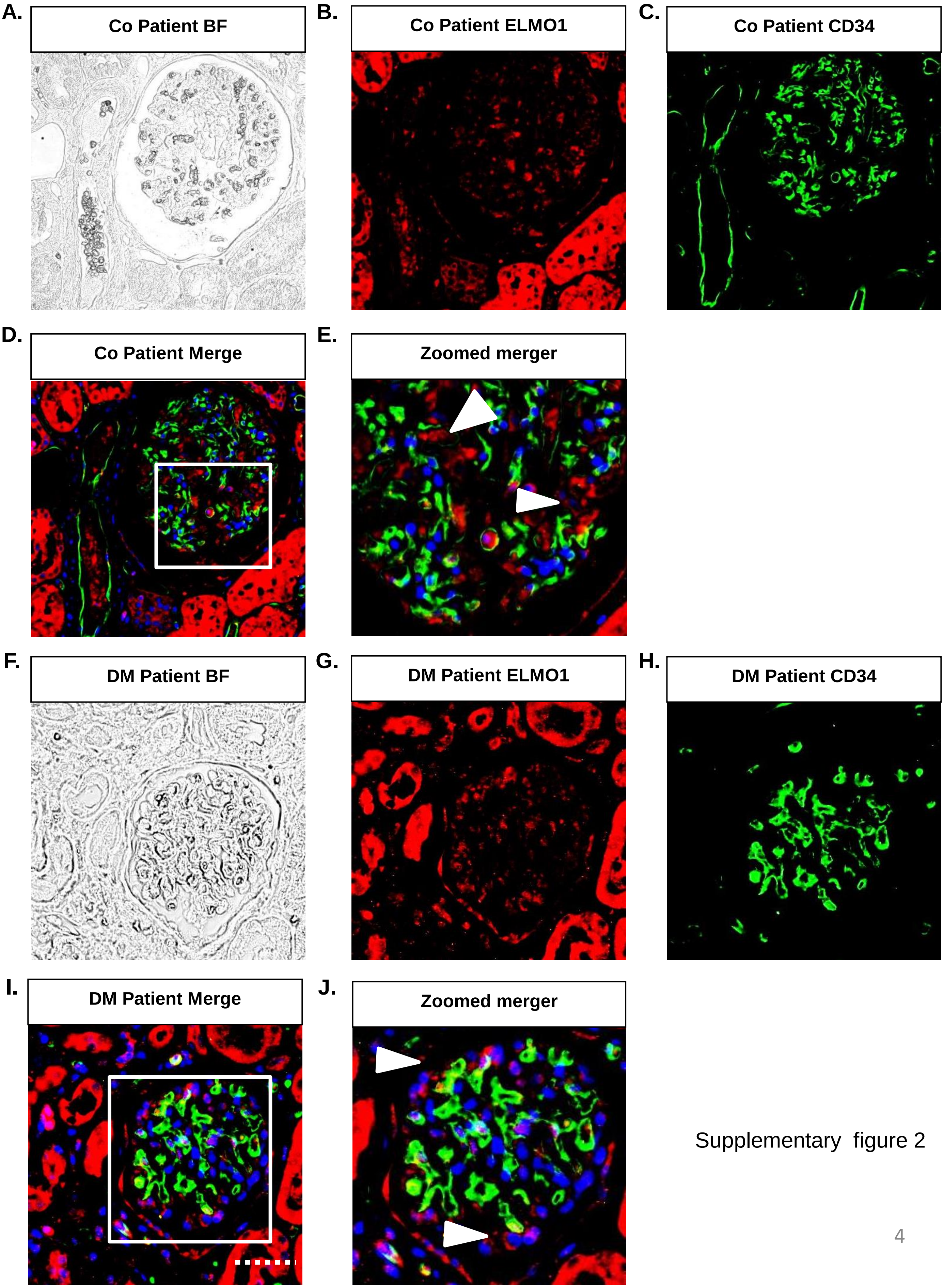


Supplementary figure 2

***Supplementary figure 2:*** **ELMO1 expression within the glomerulus is independent of endothelial cells in human control and diabetic patients. A., B., C., D.,** and **E.,** illustrate a glomerulus from a control patient. **B.** indicates ELMO1 expression, highly present in the tubules and to lesser extent within the glomerulus. **C.** illustrates CD34 (endothelial marker) expression in green. A merger of panels, **D.**, displays that ELMO1 expression within the glomerulus does not entirely co-localise with CD34 indicating that ELMO1 expression is not endothelial in origin within the renal structure. This is further emphasised in panel **E.**, where ELMO1 expression specifically in the glomerulus is indicated with white arrow heads. Similarly, **F., G., H., I.,** and **J.,** illustrate a glomerulus from a type 2 diabetic patient, displaying similar ELMO1 and CD34 expression patterns. The white dotted line in **I.**, illustrates a scale bar of 50 µm.

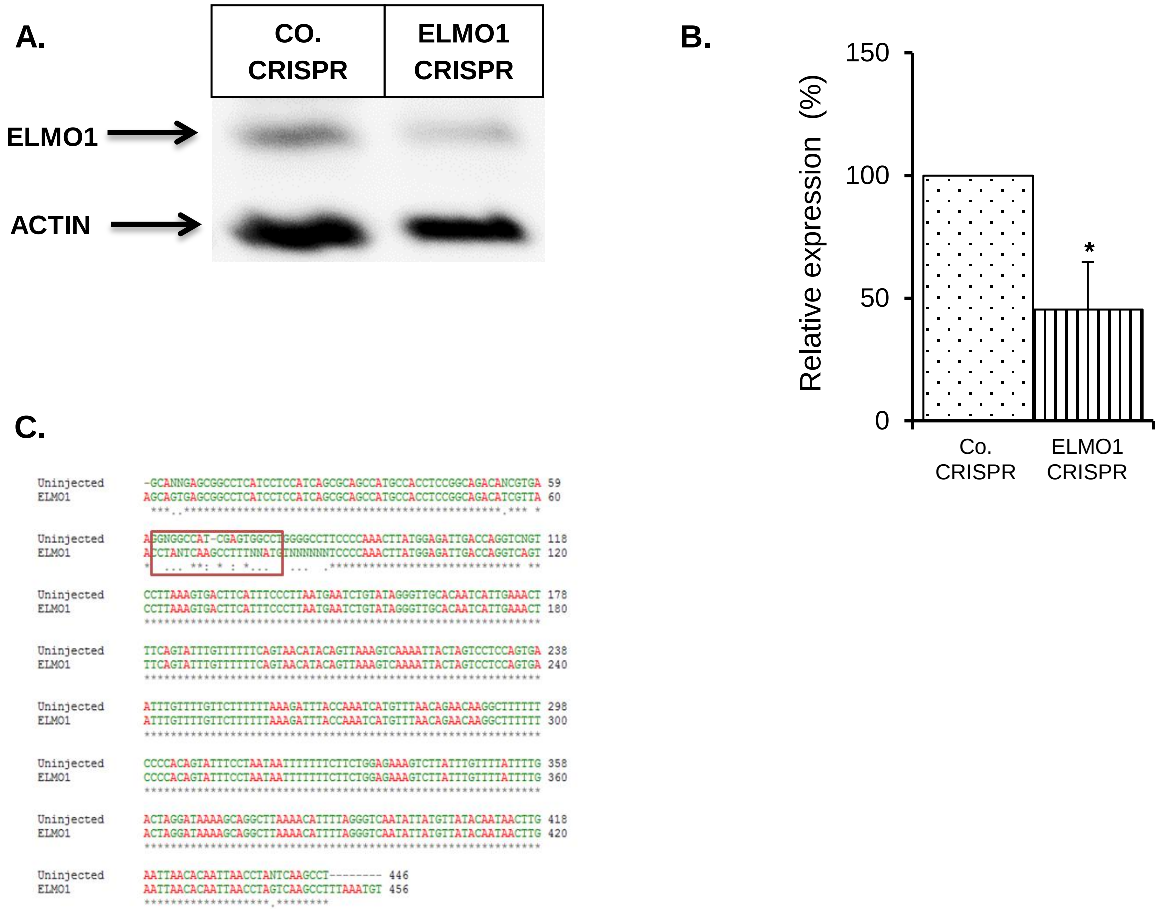


***Supplementary figure 3: Expression of ELMO1 is strongly decreased in CRISPR injected zebrafish embryos.*** **A:** Western blot analysis of zebrafish embryos upon CRISPR mediated *ELMO1* knockout, aged 48 hpf, show reduced expression of *ELMO1* as compared to the control, as seen in **A** and **B.** Each sample lane of the western blot in **A.** represent a total of 35 zebrafish embryos. The graph in **B.** illustrates the quantification of three different western blots performed, n = 3. **C.** illustrates the sequence alignment of part of the ELMO1 sequence, of a native control *Tg(wt1b:EGFP)* embryo and an ELMO1 crispant. The sequence highlighted in the red box corresponds to the ELMO1 gRNA sequence. The alignment displays the mismatches observed in ELMO1 crispants as compared to the a control.

A.

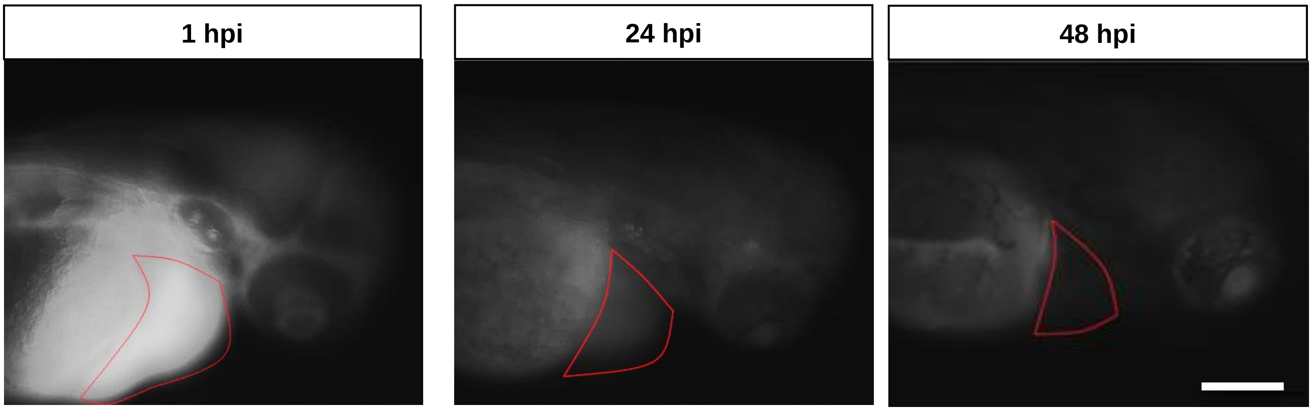


***Supplementary figure 4: Measurement of heart fluorescence over time for the pronephric functional assesment in zebrafish.*** Texas Red® tagged 70 kDa dextran was injected into the heart of ELMO1 mosaic mutants, PDX1 morphants and their respective controls, in zebrafish embryos, aged 72 hpf. Images were taken at 1 hpi, 24 hpi and 48 hpi and the relative fluorescence in the heart region, highlighted in red, was measured using imageJ. **A.** indicates the development and loss of fluorescence in an ELMO1 mosaic mutant over indicated time. The white line represents a scale bar of 200 µm.



Supplementary figure 4

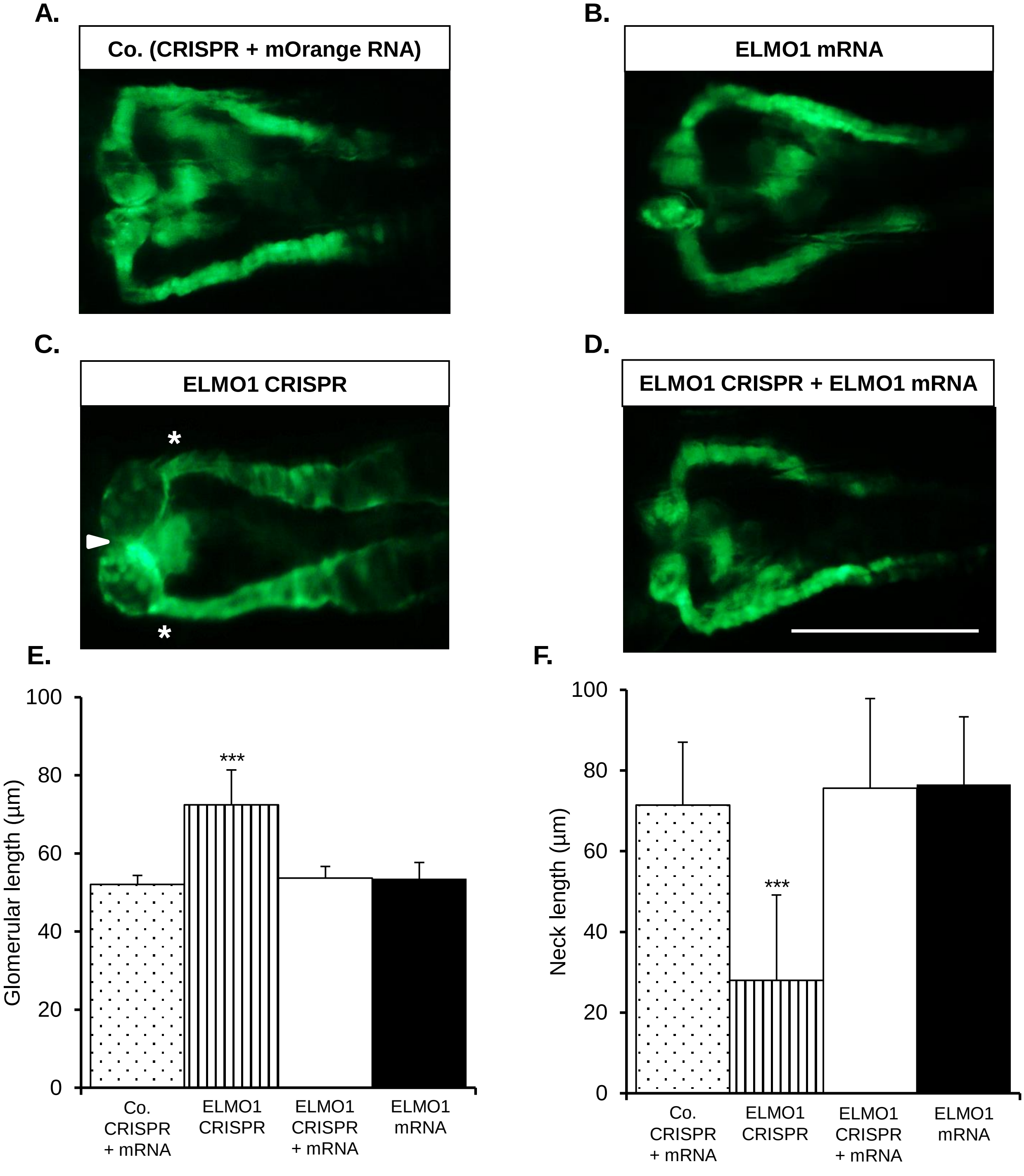


***Supplementary figure 5: Pronephric structural alterations in ELMO1 crispants could be rescued completely via ELMO1 mRNA.*** As compared to the zebrafish pronephros at 48 hpf in controls, Co. CRISPR + mRNA., **A.**, ELMO1 over-expression **B.**, has no detrimental effects on the zebrafish pronephros. **C.** ELMO1 crispants display detrimental effects on the zebrafish pronephric structure, with an enlarged glomerulus (white arrow head) and highly shortened pronephric neck (white asterix). However, **D.**, overexpression of ELMO1 in ELMO1 crispants significantly rescues pronephric phenotype. **E.**, **F.** the altered structure in ELMO1 crispants and their subsequent rescue with ELMO1 overexpression is quantified in n=40 embryos, for each condition. The white line in **D.**, indicates a scale bar of 200 µm.



Supplementary figure 5

A.

B.

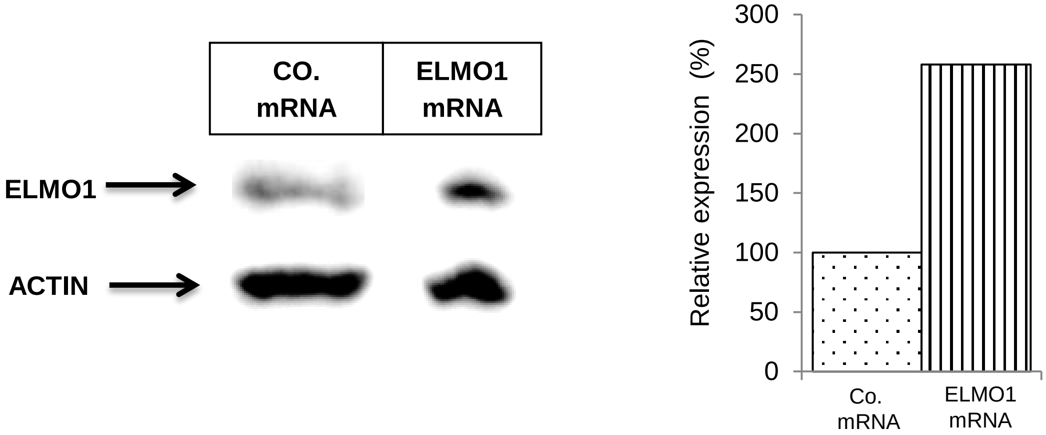


***Supplementary figure 6: Expression of ELMO1 in zebrafish embryos is increased upon ELMO1 mRNA injection.*** **A:** Western blot analysis of zebrafish embryos upon ELMO1 mRNA (300 pg) injection, aged 48 hpf, show increased expression of *elmo1* as compared to the control, as seen in **A** and **B.** Each sample lane of the western blot in **A.** represent a total of 35 zebrafish embryos. The graph in **B.** illustrates the quantification of the Western blot observed in **A**.

Supplementary figure 6

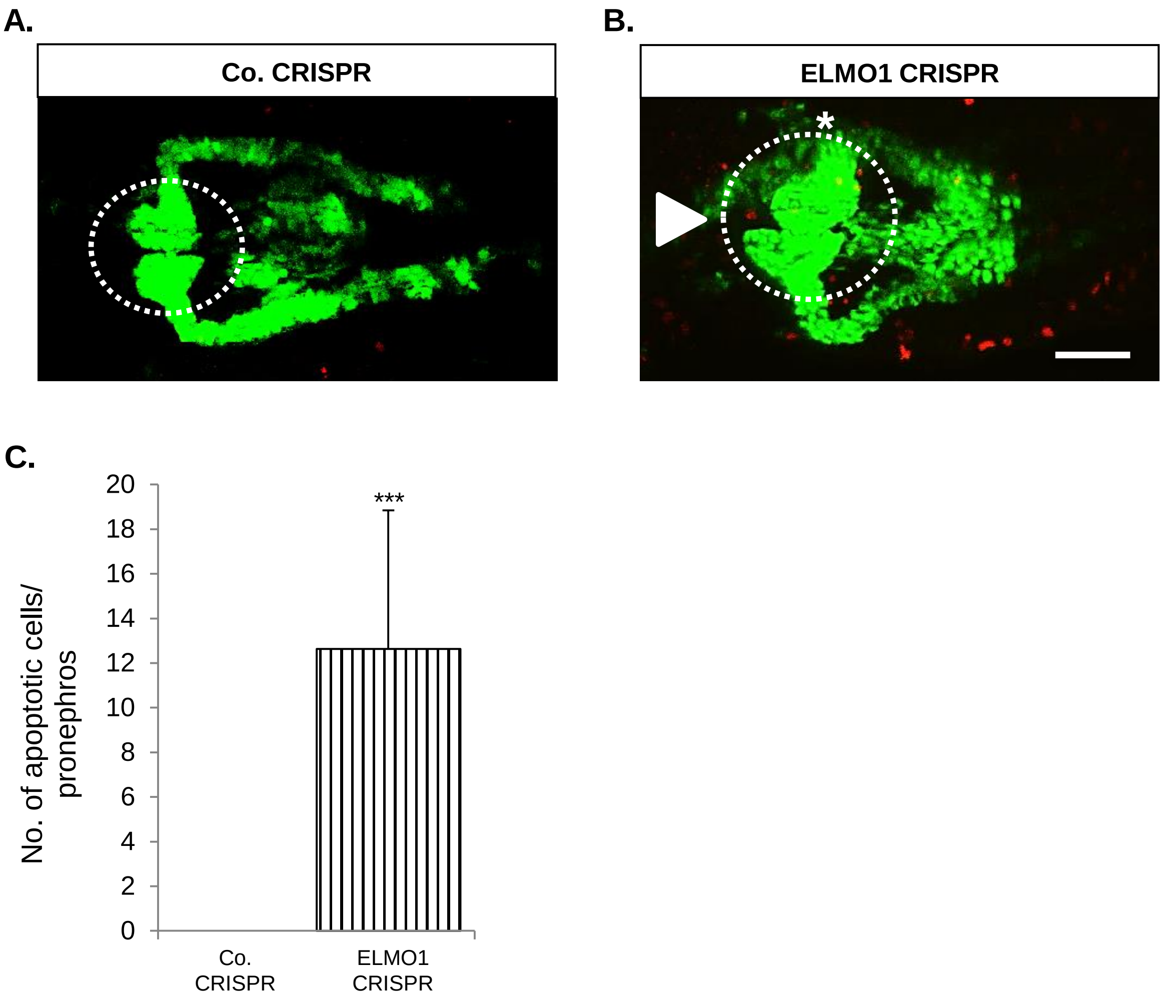


***Supplementary figure 7: CRISPR mediated knockout of ELMO1 causes an increase in apoptotic cells within the zebrafish embryo as well as the pronephros*. A.** Activated Caspase 3 assay was carried out on Co. CRISPR injected *Tg(wt1b:EGFP)* embryos at 48 hpf. The embryos showed no incidence of apoptosizing cells (indicated with the red spots) within the renal structure. **B.** ELMO1 CRISPR injected embryos showed a strong incidence of apoptosizing cells (red spots) universally as well as within the renal structure, which is quantified in the graph **C.** The number of embryos per condition analysed are 11. The white line indicates a scale bar representing 100 µm, the white arrow head indicated an enlarged glomerulus and the white asterisk illustrates shortening of the glomerular neck of the ELMO1 crispants as compared to the control. The white dotted circle encloses the glomerulus of the zebrafish pronephros.
